# Identification of metabolic biomarkers associated with nonalcoholic fatty liver disease

**DOI:** 10.1186/s12944-023-01911-2

**Published:** 2023-09-11

**Authors:** Hua Jiang, Yang Hu, Zhibo Zhang, Xujia Chen, Jianpeng Gao

**Affiliations:** Department of Gastroenterology, The Affiffiffiliated YanAn Hospital of Kunming Medical University, Kunming, China

**Keywords:** Nonalcoholic fatty liver disease, Biomarkers, Bioinformatics, Metabolism, Differentially expressed genes

## Abstract

**Background:**

Nonalcoholic fatty liver disease (NAFLD) is the most common liver disease. Metabolism-related genes significantly influence the onset and progression of the disease. Hence, it is necessary to screen metabolism-related biomarkers for the diagnosis and treatment of NAFLD patients.

**Methods:**

GSE48452, GSE63067, and GSE89632 datasets including nonalcoholic steatohepatitis (NASH) and healthy controls (HC) analyzed in this study were retrieved from the Gene Expression Omnibus (GEO) database. First, differentially expressed genes (DEGs) between NASH and HC samples were obtained. Next, metabolism-related DEGs (MR-DEGs) were identified by overlapping DEGs and metabolism-related genes (MRG). Further, a protein–protein interaction (PPI) network was developed to show the interaction among MR-DEGs. Subsequently, the “Least absolute shrinkage and selection operator regression” and “Random Forest” algorithms were used to screen metabolism-related genes (MRGs) in patients with NAFLD. Next, immune cell infiltration and gene set enrichment analyses (GSEA) were performed on these metabolism-related genes. Finally, the expression of metabolism-related gene was determined at the transcription level.

**Results:**

First, 129 DEGs related to NAFLD development were identified among patients with nonalcoholic steatohepatitis (NASH) and healthy control. Next, 18 MR-DEGs were identified using the Venn diagram. Subsequently, four genes, including *AMDHD1*, *FMO1*, *LPL,* and *P4HA1,* were identified using machine learning algorithms. Moreover, a regulatory network consisting of four genes, 25 microRNAs (miRNAs), and 41 transcription factors (TFs) was constructed. Finally, a significant increase in *FMO1* and *LPL* expression levels and a decrease in *AMDHD1* and *P4HA1* expression levels were observed in patients in the NASH group compared to the HC group.

**Conclusion:**

Metabolism-related genes associated with NAFLD were identified, containing *AMDHD1*, *FMO1*, *LPL,* and *P4HA1*, which provide insights into diagnosing and treating patients with NAFLD.

**Supplementary Information:**

The online version contains supplementary material available at 10.1186/s12944-023-01911-2.

## Introduction

Nonalcoholic fatty liver disease (NAFLD) is the hepatic manifestation of metabolic syndrome. Metabolism associated with risk factors, including obesity, hypertension, dyslipidemia, and diabetes, are associated with NAFLD [1]. NAFLD is a major cause of chronic liver disease [2]. NAFLD can be divided based on disease progression profiles into the non-alcoholic fatty liver (NAFL), nonalcoholic steatohepatitis (NASH), and liver fibrosis as well as cirrhosis [3]. Studies have shown that the incidence of NAFLD among adults in European and American countries is 20%-33% [4]. NAFLD has overtaken viral hepatitis as a major chronic liver disease. Moreover, the incidence of NAFLD is increasing year by year and becoming younger [3]. Globally, NAFLD is the most prevalent type of chronic liver disease [5]. Although treating patients with NAFLD at an early stage could help in complete recovery, approximately 20% of patients with NAFLD progress to cirrhosis and end-stage liver disease [4]. This seriously endangers the health of people [6]. Additionally, NAFLD is involved in the onset and progression of diabetes, arteriosclerosis, and several chronic liver diseases, respectively, seriously affecting the quality of life and life expectancy [7]. Due to the regulation of insulin resistance, there is an increased risk of developing cancers other than the liver, such as bladder cancer [8]. Therefore, identifying biomarkers for diagnosing patients with NAFLD is the need of the hour.

Metabolic dysfunction, such as hepatic steatosis, is an early indicator of NAFLD development. Studies have shown the interaction between metabolic pathways, intestinal flora, and immune systems in patients with NAFLD. Immune disorders, intestinal imbalances, and metabolic disorders promote liver inflammation and other aspects during late-stage NAFLD [9]. A study has shown that inflammation and metabolic processes in adipose tissue could accelerate NASH development and serve as therapeutic targets [10]. In fact, some studies have suggested that NAFLD could be referred to as metabolic (dysfunction) associated fatty liver disease (MAFLD) [11]. Therefore, determining the influence of metabolism on regulating NAFLD would aid in designing targeting therapies [12]. However, the role of metabolism-related genes (MRGs) in NAFLD is yet to be elucidated.

In this study, publicly available databases were searched, followed by bioinformatic analysis to identify MRGs in NAFLD onset and progression. Next, the regulatory network of MRGs and their correlation with the immune microenvironment were analyzed. The results of this research would aid in understanding the mechanisms of MRGs in NAFLD and identifying new therapeutic targets.

## Materials and methods

### Data acquisition

The GSE48452 and GSE63067 datasets consisting of data on clinical features and gene expression in liver tissue of patients with NASH and healthy controls (HC) were retrieved from the Gene Expression Omnibus (GEO) database based the source and size of samples. GSE48452 comprised 14 HC and 18 patients with NASH, and GSE63067 comprised seven HC and nine patients with NASH. Additionally, the GSE89632 dataset, comprising 24 HC and 19 patients with NASH, was used to verify the expression level of biomarkers. Finally, based on the background gene set “c2.cp.kegg.v7.4.symbols.gmt”, the gene set related to metabolism were obtained from The Molecular Signatures Database (MsigDB) database (v7.4, https://www.gsea-msigdb.org/gsea/msigdb/), and 948 metabolism-related genes (MRGs) were acquired by merging the genes from gene set and after de-duplicating. The flow chart of this study is shown in Supplementary Fig. 1.

### Removing batch effects

To minimize the effects of sequencing platforms, experimental environments, sample processing and other factors, the Combat function in the “sva” package [13]was used to implement the de-batch of GSE48452 and GSE63067 datasets. Then, the merged dataset was used as a training cohort. Furthermore, the “ggplot2” package and UMAP algorithm were used to validate the correction results.

### Analysis of differential genes

First, the “linear models for microarray data” R package [14] were used to identify differentially expressed genes (DEGs) based on “*P* < 0.05” and “|log_2_fold change (FC)|> 0.5” criteria in the NASH and HC groups in the training cohort. Next, a volcano plot and heatmap were constructed to show DEGs in NAFLD. Metabolism-related DEGs (MR-DEGs) were identified by intersecting DEGs and key module genes using the “VennDiagram” package [15].

### Functional enrichment analysis and Protein–protein interaction (PPI) network

Gene Ontology (GO) and “Kyoto Encyclopedia of Genes and Genomes (KEGG) pathway enrichment analyses were conducted on MR-DEGs using the “clusterProfiler” package [16] with “*P* < 0.05” as screening criteria. Next, a PPI network was constructed to present the interaction between MR-DEGs using the STRING (https://string-db.org) database. Finally, the topological features of the network were visualized using the “Cytoscape (version 3.7.2)” software [17].

### Machine learning methods

The “Least absolute shrinkage and selection operator (LASSO)” regression analysis was performed, and the random forest (RF) algorithm was implemented using the “randomForest” package to screen important genes in the training cohort. The LASSO algorithm was performed by “glmnet” package [18] (version4.0–2), with parameters set to: family = binomial, type.measure = class, nfold = 10, to reduce the feature dimensions. Biomarkers were identified by intersecting genes identified using these algorithms. Moreover, the diagnostic significance of these genes was evaluated by constructing receiver operating characteristic (ROC) curves using the “pROC” package [19]. GSE89632 served as the external validation cohort for verifying these biomarkers. Based on the expression of biomarkers and sample grouping information in the training set, the nomogram was established by “RMS” [20] package (version6.4–1). To assess the predictive power of the nomogram, the calibration curve was plotted and the consistency index calculated using the calibrate function in the “RMS” package.

### Immune cell infiltration (ICI) and Gene set enrichment analysis (GSEA)

The single-sample GSEA (ssGSEA) algorithm was used for calculating the relative abundance of 28 immune cells infiltrating the immune microenvironment of patients with NAFLD. Next, the correlation between diagnosis-related MRGs and differentially infiltrating immune cells was calculated and visualized. Finally, GSEA was performed to identify MRG-enriched KEGG pathways and GO terms using the “clusterProfiler” package [21].

### Constructing biomarker regulatory networks

Biomarker-related micro-RNAs (miRNAs) were screened using the miRwalk3.0 database as per the following criteria: “binding probability ≥ 0.95” and “binding site position at 3’ untranslated region (UTR).” In addition, the transcription factors (TFs) targeting these biomarkers were predicted using the “hTFtarget” database. Finally, the TF-miRNA-mRNA network was constructed with the aid of the “Cytoscape (version 3.8.2)” software.

### Analysis of MRG expression

Quantitative reverse transcriptase PCR (RT-qPCR) was performed to determine MRG expression profiles among patients with NAFLD. Blood samples were collected from five HC and five patients with NASH. Liver tissue and serum samples were collected from patients undergoing liver biopsy between April 2019 and July 2020, and categorized based on histopathological diagnosis into hepatic steatosis (HS), non-alcoholic steatohepatitis (NASH), and hepatitis control (HC). Participants with predetermined abnormal imaging parameters were offered a liver biopsy. Biopsies were read in a blinded fashion with results based on the consensus by 2 expert pathologists.The prevalence of NASH was defined by biopsy. The demographic information of the included participants was shown in Supplementary Table 1.

All participants provided informed consent to participate in the study. This study was approved by the Medical Ethics Committee of Yan'an Hospital, affiliated with the Kunming Medical University ethics committee. First, total RNA was isolated from ten blood samples using TRIzol (Ambion, Austin, USA). Next, cDNA was synthesized by reverse transcribing total RNA into cDNA using the First-strand-cDNA-synthesis-kit (Servicebio, Wuhan, China). Finally, qRT-PCR was performed using the 2xUniversal Blue SYBR Green qPCR Master Mix (Servicebio, Wuhan, China). All these experiments were performed based on the protocol provided by the manufacturer. The sequences of primer used for PCR were designed based on the length of primer (17–25 bp), Tm value (58–62℃), GC content (40–60%), the size of the product (100–200 bp) and so on (Table 1). *GAPDH* served as an internal reference gene. Gene expression was calculated using the 2-ΔΔCt method [22].

**Table 1:** Gene-specific primer sequences used for qRT-PCR

Primer	Sequence
*AMDHD1*-F	GGGATGAACTCCACCCGATG
*AMDHD1*-R	CGATCCGTGTGTGTGAGACT
*FMO1*-F	AGAGAACATGGCCAAGCGAG
*FMO1*-R	TTCGGTGAATCTCCACAGCC
*LPL*-F	AAGGCCTACAGGTGCAGTTC
*LPL*-R	CCAGATTGTTGCAGCGGTTC
*P4HA1*-F	AATGACCCCTCGGAGACAGA
*P4HA1*-R	TGGCTCATCTTTCTGTAATTCCTCT
*GAPDH*-F	CGAAGGTGGAGTCAACGGATTT
*GAPDH*-R	ATGGGTGGAATCATATTGGAAC

## Results

### Subject characteristics

The demographic and clinical characteristics of the participants are summarized in supplementary Table 1. There were no significant differences in age, sex, race, diabetes and hypertension prevalence between the NASH and HC groups. The levels of TC and BMI in NASH group were higher than those in HC group, but there was no significant difference in TG, LDL-C, HDL-C, ALT, AST, GGT and WC between the two groups.

### Identifying MR-DEGs in Patients with NAFLD

**Fig. 1:**
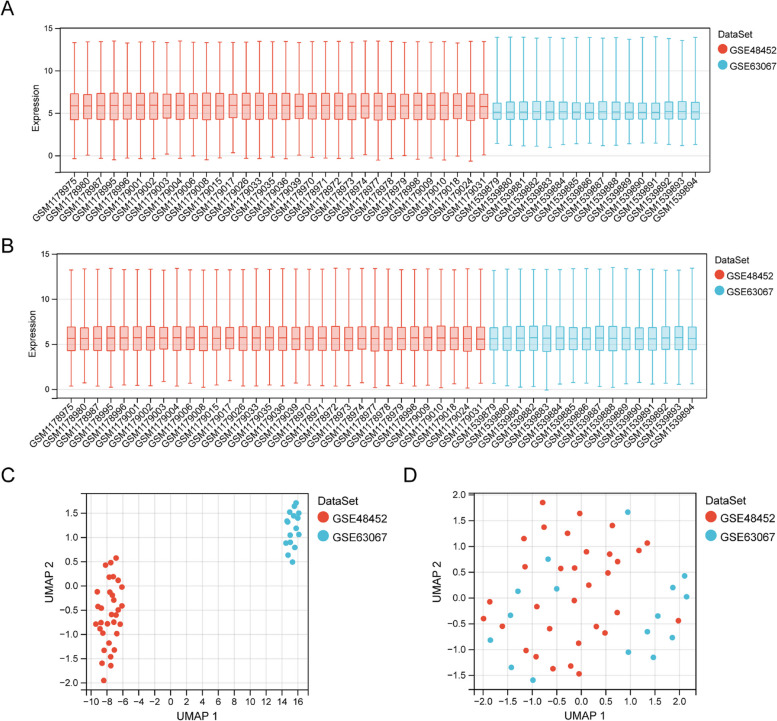
Eliminating batch effects in GSE48452 and GSE63067 datasets. **A**, **B** The boxplots show two datasets before (**A**) and after (**B**), eliminating batch effects. **C**, **D** Principal component analysis (PCA) plots of two datasets before (**C**) and after (**D**) eliminating batch effect

**Fig. 2:**
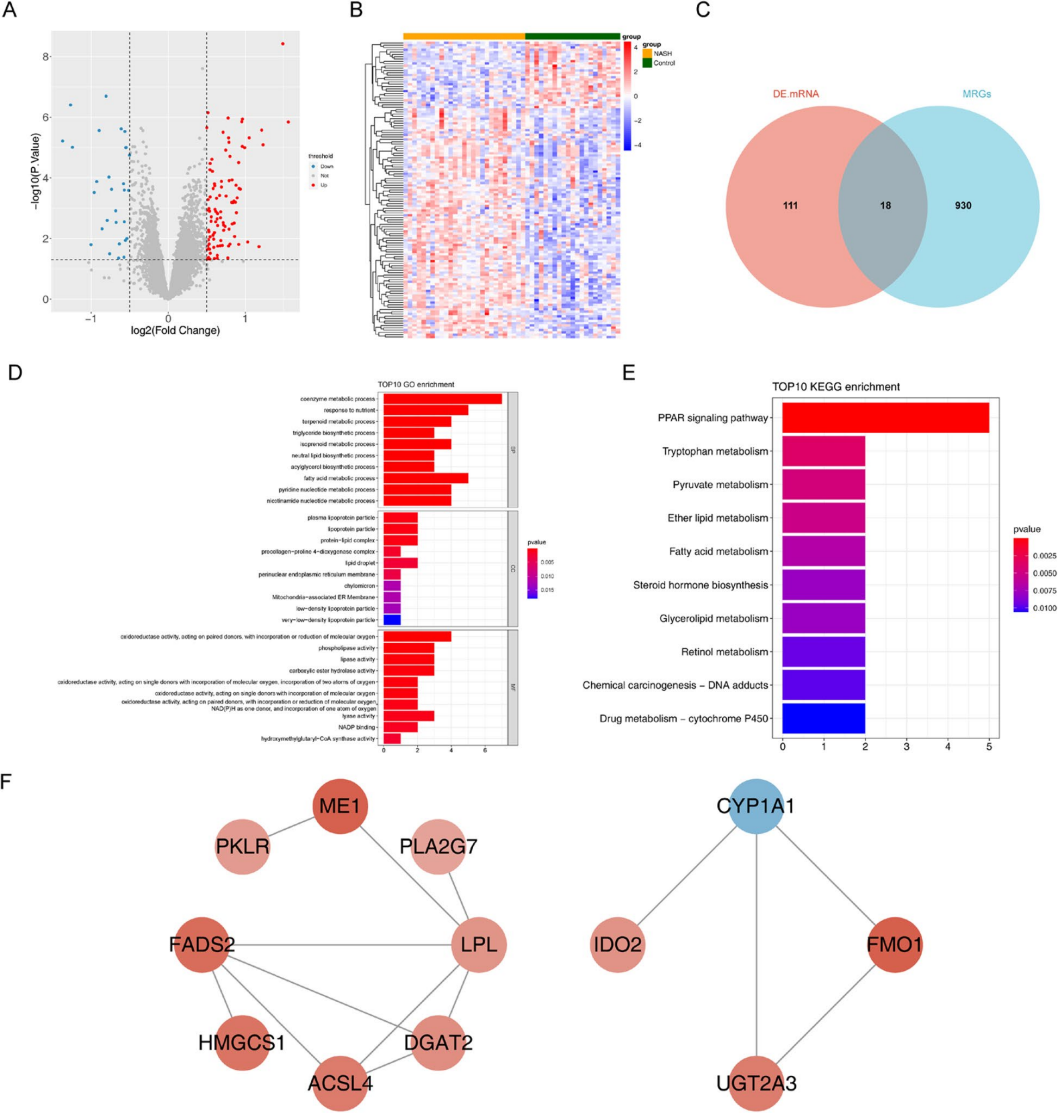
Identifying metabolism-related differentially expressed genes (MR-DEGs) and functional enrichment analysis. **A**, **B** The volcano map (**A**) and heat map (**B**) show 129 differentially expressed genes (DEGs) between the nonalcoholic steatohepatitis (NASH) and healthy controls (HC) groups in the merged dataset. **C** The Venn diagram of 18 MR-DEGs was obtained by overlapping DEGs and 948 metabolism-related genes (MRGs). **D**, **E** Gene ontology (GO) terms and Kyoto Encyclopedia of Genes and Genomes (KEGG) pathways enriched by MR-DEGs. BP, biological progress; CC, cellular component; MF, molecular function. **F** The protein–protein interaction (PPI) network of MR-DEGs

Principal component analysis (PCA) plots show GSE48452 and GSE63067 before and after eliminating batch effects between datasets (Fig. 1A-D). In total, 129 DEGs among patients with NASH and HC in the merged dataset were identified. Of which 100 genes were upregulated, and 29 genes were downregulated genes (Fig. 2A). These DEGs were visualized using a heatmap (Fig. 2B). Finally, DEGs and 948 MRGs were overlapped to identify 18 MR-DEGs (Fig. 2C). Subsequently, functional enrichment analysis was performed to identify the underlying mechanisms of NAFLD-associated MR-DEGs. Figure 2D shows the top ten GO terms under each classification. The results revealed that these MR-DEGs were primarily enriched in the “isoprenoid metabolic process,” “acylglycerol biosynthetic process,” and “fatty acid metabolic process.” The KEGG pathways enriched by these MR-DEGs primarily included the “biosynthesis of steroid hormones” as well as the “fatty acid and glycerolipid metabolism” pathways (Fig. 2E). Finally, a PPI network of MR-DEGs was constructed, which included 12 nodes (11 up-regulated genes and 1 down-regulated genes) and 14 edges (Fig. 2F). The PPI network revealed that *LPL* had interactions with *PLA2G7*, *ME1*, *FADS2*, *ACSL4*, and *DGAT2*. Meanwhile, *FMO1* interacted with *CYP1A1* and *UGT2A3*.

### Screening of biomarkers related to metabolism

**Fig. 3:**
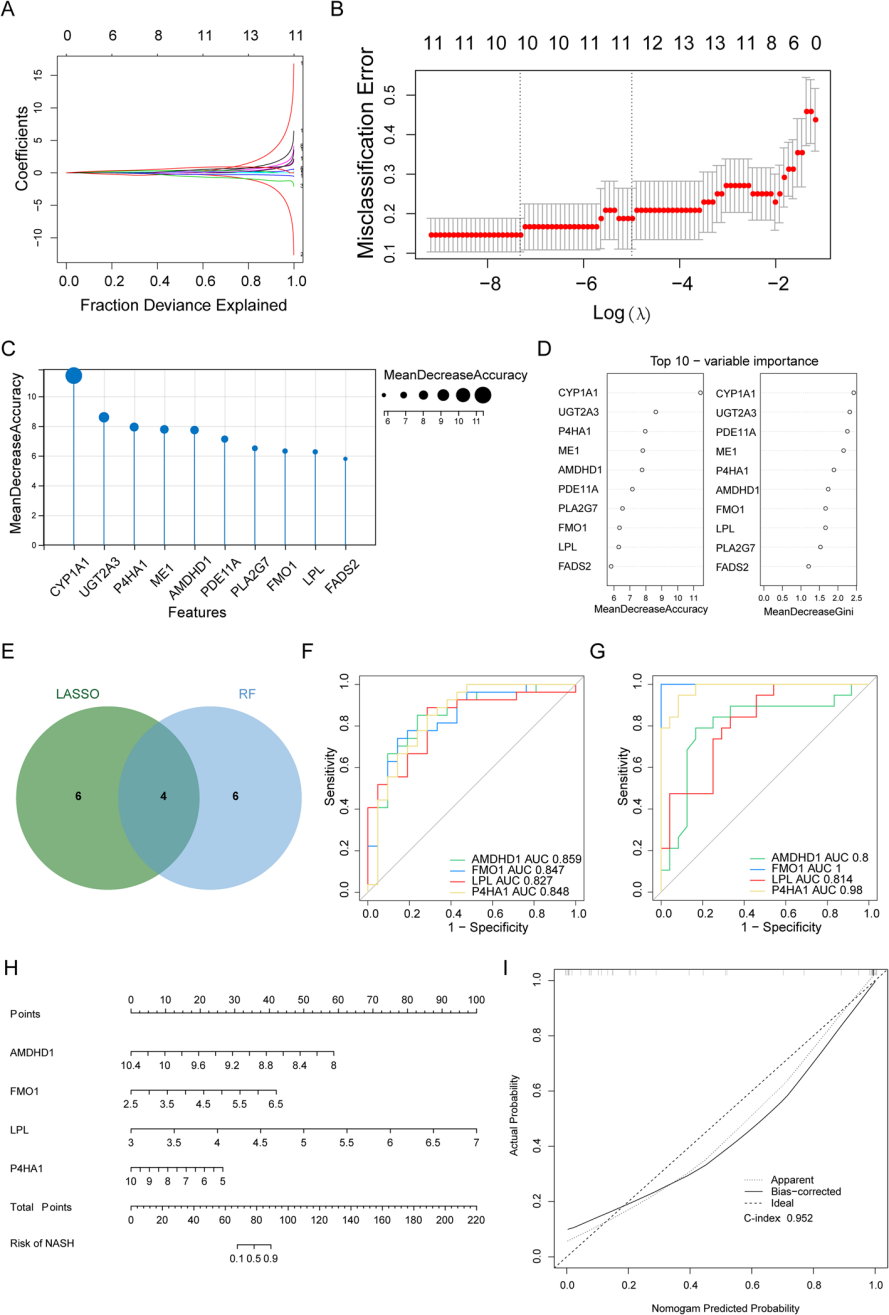
Identification of biomarkers for non-alcoholic fatty liver disease (NAFLD). **A**, **B** 18 key genes were screened using least absolute shrinkage and selection operator (LASSO). **C**, **D** Ten feature genes were retained by the random forest (RF) algorithm. **E** The Venn diagram of four biomarkers. **F**, **G** The receiver operating characteristic (ROC) curves of biomarkers in the training and external validation sets. AUC, the area under the curve. **H** The nomogram of biomarkers. **I** The calibration curve of the nomogram

To further dig out the key genes, LASSO regression analysis was performed on 18 MR-DEGs to screen for key genes, and 10 feature genes were identified, including *ACSL4*, Amidolytic domain1 (*AMDHD1)*, *CA14*, *DGAT2*, Flavin-Containing Dimethylaniline Monooxygenase 1 (*FMO1)*, IDO2, Lipoprotein lipase (*LPL)*, (Proline 4-hydroxylase subunit α1) *P4HA1*, *PKLR*, and *TREH* (Fig. 3A-B). Meanwhile, 10 feature genes were identified, including *CYP1A1*, *UGT2A3*, *P4HA1*, *ME1*, *AMDHD1*, *PDE11A*, *PLA2G7*, *FMO1*, *LPL*, and *FADS2* using the RF algorithm (Fig. 3C-D). Subsequently, four overlapping genes, *AMDHD1*, *FMO1*, *LPL*, and *P4HA1,* were obtained using these algorithms (Fig. 3E) and defined as metabolism-related biomarkers (MRBs) in NAFLD. The AUC values of the MRBs were > 0.8, thus indicating that these MRBs had good diagnostic accuracy (Fig. 3F). Next, the diagnostic value of biomarkers in the external validation set (GSE89632) was further validated, and the results were consistent with the training set (Fig. 3G). Subsequently, the nomogram (C-index = 0.95) was created to further explore the clinical value of biomarkers (Fig. 3H). The calibration curve showed that the error between the actual and predicted risk was small, indicating that the nomogram model has high prediction accuracy for NASH samples (Fig. 3I).

### Immune infiltration and functional enrichment analysis

**Fig. 4:**
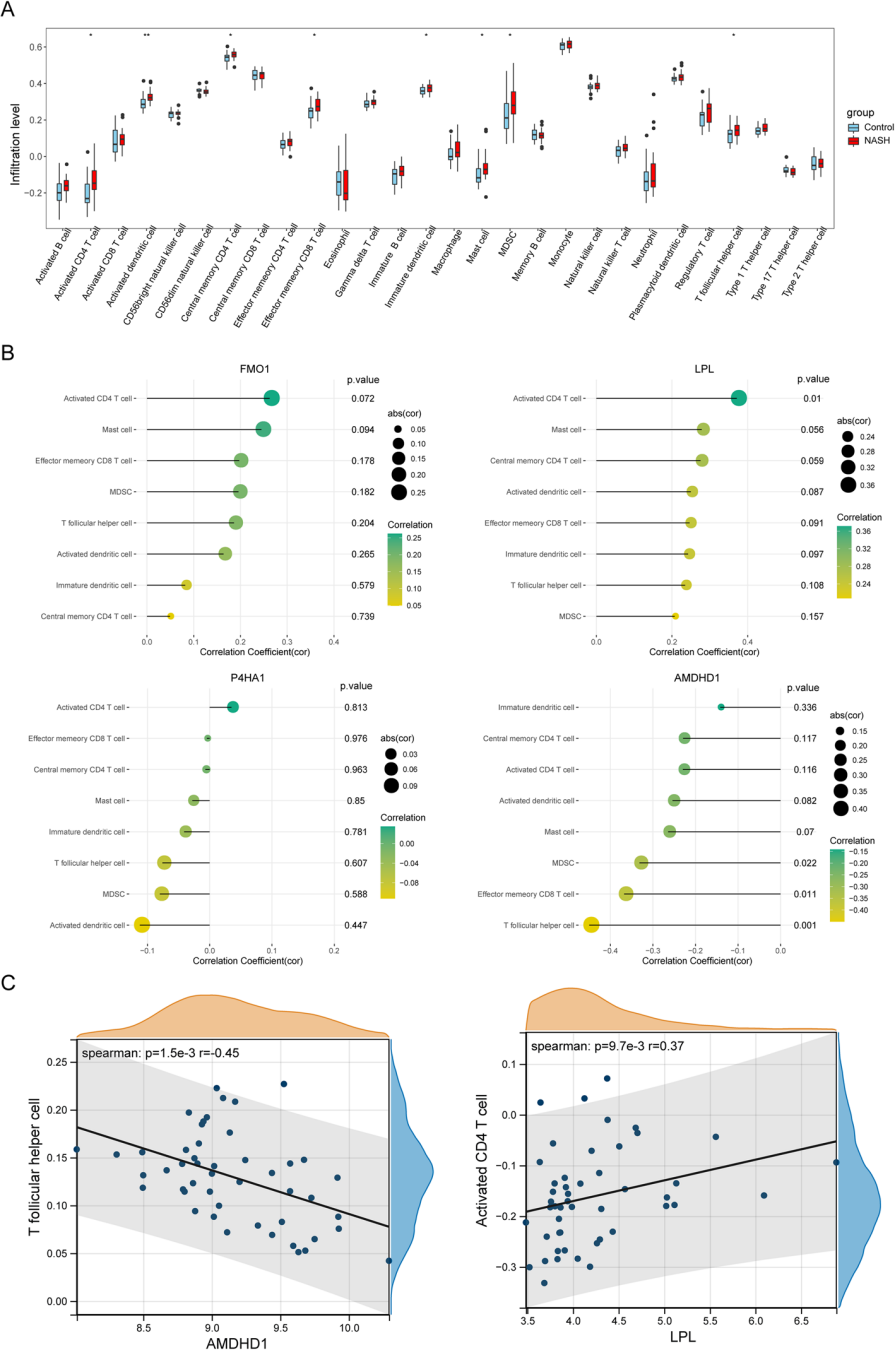
Immune infiltration and functional enrichment analyses. **A** The relative abundance of 28 immune cells in the immune microenvironment of patients with NAFLD. **B** The correlation between MRBs and differential immune cells. **C** Correlation between hub gene expression and differential immune cells in patients with NAFLD

To analyze the immune microenvironment of patients with NAFLD, the relative abundance of 28 immune cell types was compared between both groups in the training cohort. The results revealed significant differences in the abundance of eight immune cells, including activated and central memory CD4 T cells, immature and activated dendritic cells (DCs), effector memory CD8 T cells, mast cells, myeloid-derived suppressor cells (MDSCs), and T follicular helper (Tfh) cells (Fig. 4A). Figure 4B shows the correlation between MRBs and differential ICI. A significant negative correlation between *AMDHD1* and Tfh cells as well as a positive correlation between *LPL* and activated CD4 T cells was observed (Fig. 4C). Next, ssGSEA was performed on biomarkers to determine the possible functions of *AMDHD1*, *FMO1*, *LPL*, and *P4HA1* in NAFLD. The results showed that *AMDHD1* was mainly enriched in the ‘cytoplasmic translation’ and ‘ECM − receptor interaction’ (Supplementary Fig. 2A). Additionally, *FMO1* was primarily enriched in the intrinsic apoptotic and FoxO signaling pathways (Supplementary Fig. 2B). “Chromosome segregation” and “complement and coagulation pathways” were enriched primarily by *LPL* (Supplementary Fig. 2C). *P4HA1* was primarily enriched in the “organic acid catabolic process” and “the interactions between neuroactive ligand receptors” (Supplementary Fig. 2D).

### Four MRB-based regulatory network

**Fig. 5:**
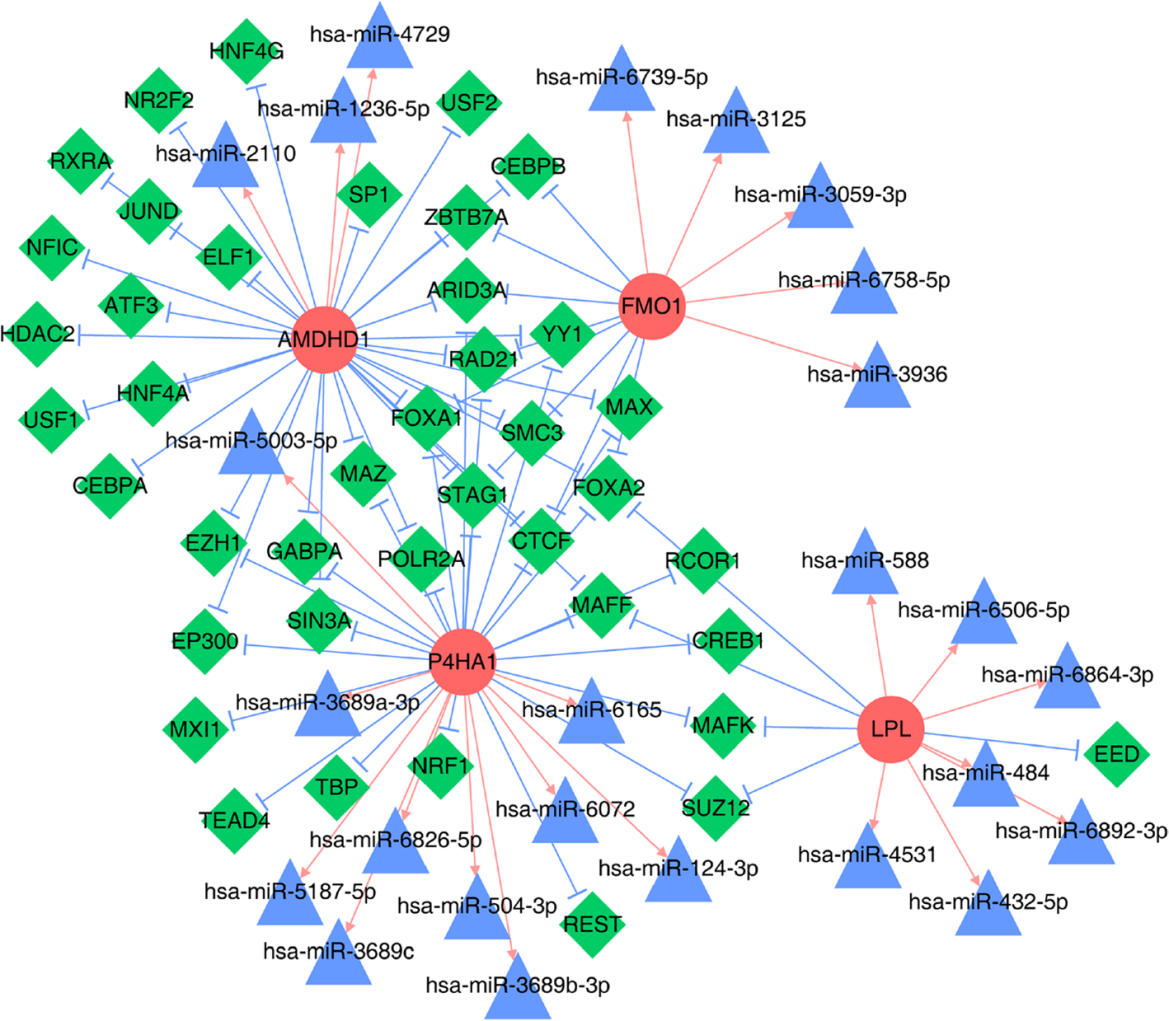
The regulatory network of four biomarkers. Red circles represent biomarkers; green diamonds represent transcription factors (TFs); blue triangles represent microRNAs (miRNAs); pink arrowheads represent miRNA-mRNA pairs, and blue T-shaped lines represent TF-mRNA pairs

A regulatory network integrating *AMDHD1*, *FMO1*, *LPL*, and *P4HA1* was constructed to identify the underlying regulatory mechanism of these MRBs. In addition, mRNA-miRNA and TF-mRNA pairs were matched to construct an “mRNA-miRNA-TF” regulatory network consisting of 25 miRNAs, four mRNAs, 41 TFs, 37 nodes, and 51 edges (Fig. 5). The results revealed that *AMDHD1*, *P4HA1*, and *FMO1* could have common regulatory factors, such as PAD21 and CTCF.

### Validating MRB expression

**Fig. 6:**
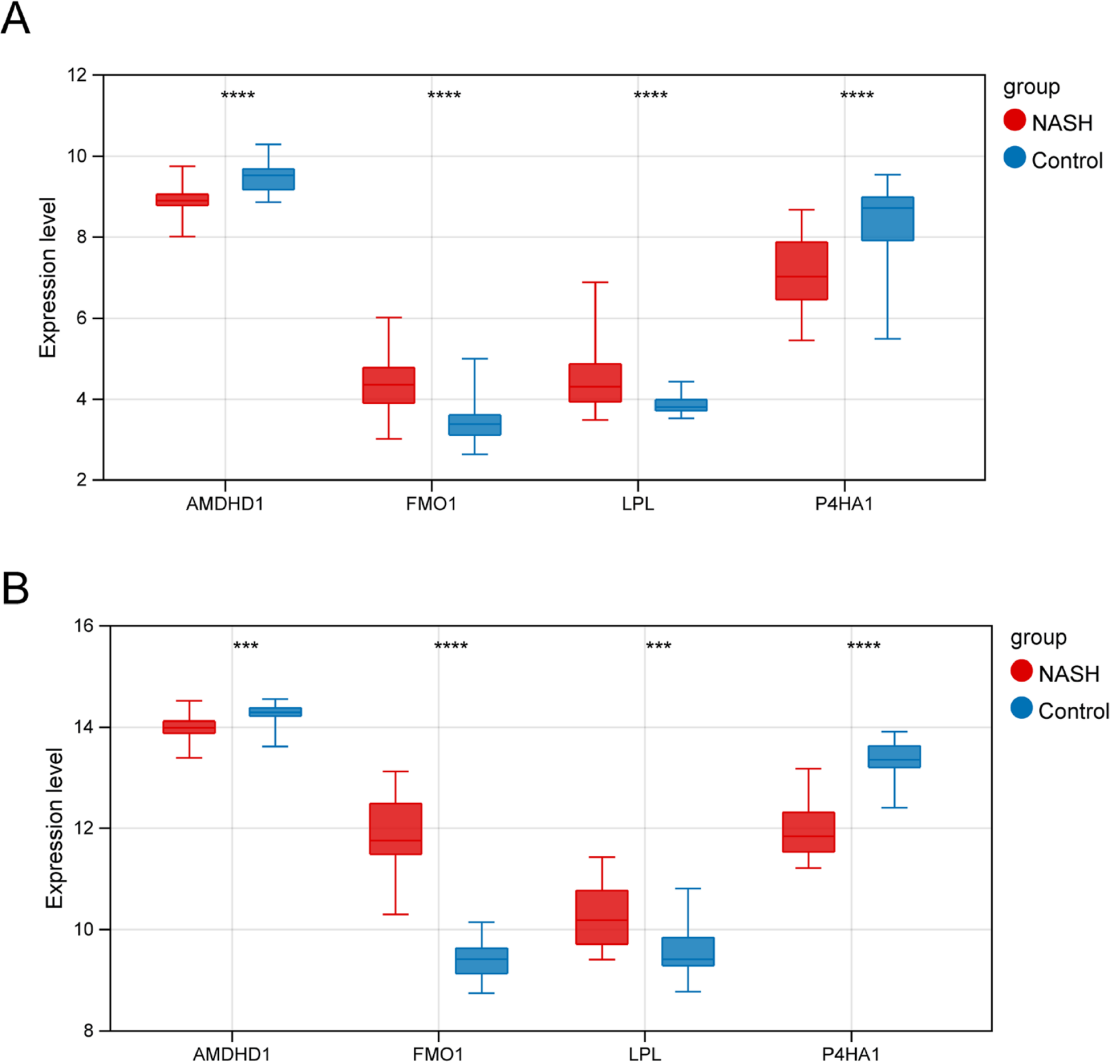
The expression of biomarkers in the training set (**A**) and GSE89632 (**B**). *** *P* < 0.001, **** P < 0.0001

**Fig. 7:**
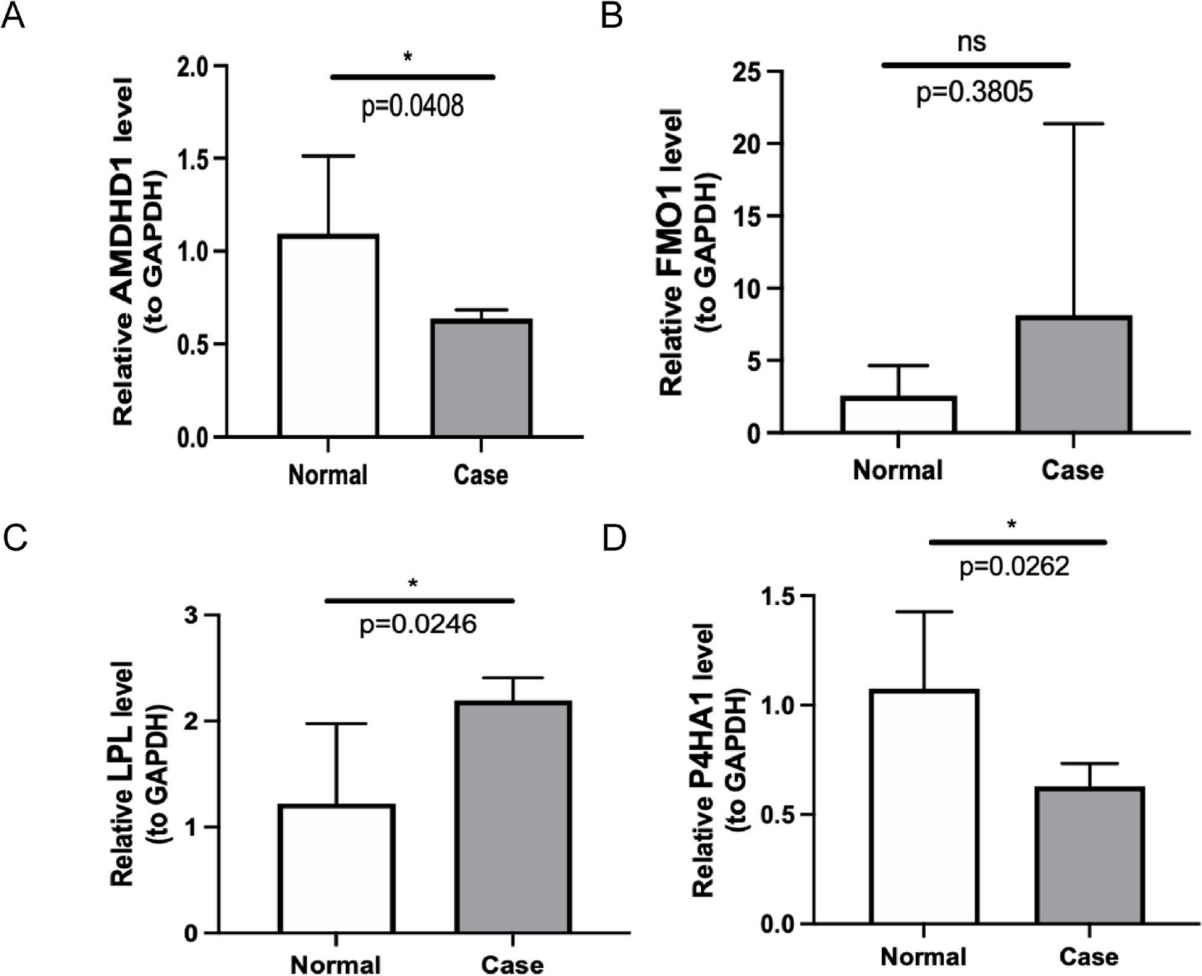
Validating the expression of biomarkers using reverse transcription quantitative PCR (RT-PCR). ns, not significant; *P < 0.05

Prognostic gene expression in patients in the training set and GSE89632 was determined, respectively. The results revealed an increase in *FMO1* and *LPL* expression levels and a decrease in *AMDHD1* and *P4HA1* expression levels among patients in the NASH group compared to the HC group in the training cohort (Fig. 6A). In GSE89632, a significant decrease in *AMDHD1* and *P4HA1* expression level and a significant increase in *FMO1* and *LPL* expression level were observed in patients in the NASH group compared to the HC group (Fig. 6B). Finally, the expression of these MRBs in the blood samples of the patients was determined using qRT-PCR. The results were consistent with the results obtained by analyzing publicly available databases (Fig. 7).

## Discussion

Adipocyte dysfunction affects NAFLD onset and progression and could be a therapeutic target for treating patients with NAFLD. However, there were significant challenges in addressing the global epidemic of NAFLD, metabolic, and hepatic complications [23]. In this study, four NAFLD-associated MRBs were identified to determine the correlation between MRGs and differential ICI.

*AMDHD1* is localized in the cytoplasm, which catalyzes the non-peptide carbon–nitrogen bond hydrolysis in cyclic amides. *AMDHD1* encodes a 426 amino acid protein. It is involved in the catabolism of histidine, including glutamic acid and formamide, glutamic acid, and Formate [24]. *AMDHD*1 is highly expressed in liver-specific [25]. A study showed a significant increase in *AMDHD1* expression level in the regenerating liver compared to the developing liver, thereby indicating that *AMDHD1* is involved in the renewal and repair of the liver [26]. Our results revealed a decrease in *AMDHD1* expression level in patients with NAFLD, which suggest inhibition in the regeneration and development of the liver among patients with NAFLD. The failure of liver tissues to adequately repair after damage due to inflammation leads to NAFLD onset and progression. A study performed with integrated proteomics and bioinformatic analysis showed that *AMDHD1* could predict the prognosis of patients with hepatocellular carcinoma (HCC) [27].

FMOs are involved in the metabolism of small-molecule drugs. In humans, five FMOs, such as FMO1, FMO3, and FMO5, are involved in drug metabolism in the liver. FMO1 and FMO3 convert Trimethylamine N-oxide (TMAO), a metabolite, to trimethylamine in the liver [28]. Because it was associated with insulin resistance, the formation of atherosclerotic plaques, cancer, diabetes, heart failure, and Hepatic steatosis was positively correlated. TMAO is produced by the fermentation of dietary nutrients, such as choline and carnitine, in the gut microbiota. In metabolic syndromes, an increase in TMAO levels occurs along with an increase in body mass, visceral adiposity, and fatty liver indices [29]. Our results revealed that *FMO1* is a hub gene in NAFLD; however, the involvement of *FMO1* in NAFLD pathogenesis is yet to be determined. A study used marker-free quantitative proteomics to demonstrate the effect of NASH on biological processes and functions in rats. The results showed a decrease in *FMO1* expression level in rats in the NASH group, indicating significant involvement of *FMO1* in NAFLD progression [30].

*LPL* encodes for lipoprotein lipase, detected in cardiac, muscle, and adipose tissue. LPL is a homodimer and acts as a triglyceride hydrolase and a ligand-bridging factor involved in the uptake of lipoproteins mediated by the receptors. LPL binds to glycerol tripolyester. It participates in catalysis, the process associated with changes in cellular state or activity (such as motility, secretion, enzyme production, gene expression, etc.), and the internal homeostasis of cholesterol. Gain, loss, or modification of proteins or lipids in chylomicrons, including lipoprotein lipase hydrolysis of triglycerides and subsequent loss of free fatty acids. LPL activates or enhances the frequency, rate, or degree of adipocyte differentiation. Severe mutations due to LPL deficiency cause type I hyperlipoproteinemia. In fact, LPL mutations are involved in multiple lipoprotein metabolism diseases. Moreover, rs328 polymorphism in the G allele of *LPL* could reduce the risk of abdominal obesity [31]. Fibrin and omega-3 fatty acids reduce TG levels. However, ideal therapeutic strategies for patients with high TG and TGRL levels and low HDL levels are still unavailable. LPL is a key regulator of lipids. In lipoprotein particles, lipids into glycerol and free fatty acids are hydrolyzed by LPL for the storage and consumption of lipids by peripheral organs. With an enhancement in our comprehension of human genetics, proteins regulating LPL activity, including the apolipoprotein and angiopoietin-like families, have been identified.

A previous study showed an increase in *LPL* expression levels among patients with NASH, consistent with our results. *LPL* is critically involved in incorporating plasma lipids into tissues, regulates the metabolism of lipids, and energy balance in the body [32]. The hepatic stellate cells (HSC) activation pathway mediated by LPL could be targeted for treating liver fibrosis in patients with NASH [33]. During fasting, high insulinemia and blood glucose could reduce the LPL-mediated catabolism of triglyceride-rich lipoproteins and increases postprandial levels [34]. LPL regulates lipid metabolism by hydrolyzing triglycerides and very low-density lipoproteins in chylomicrons. In high-fat diet-fed mice, a high *LPL* expression level could attenuate lipid droplet accumulation in the liver and improve the metabolism of glucose. These results could aid in designing new drugs to treat metabolic syndromes, such as type 2 diabetes and NAFDL [35]. Studies have shown that targeting the LPL/FABP4/CPT1 axis could be a promising strategy for preventing NASH-related HCC [36].

*P4HA1* encodes for a component of proline 4-hydroxylase, a key enzyme in the synthesis of collagen and composed of two. The protein encoded by *P4HA1* contains several different alpha subunit types and serves as a major portion of the catalytic site of an active enzyme. *P4HA1* also acts as a cofactor and antioxidant in several species [37]. *P4HA1* is an essential rate-limiting enzyme and a P4H (also called PHD) isoenzyme [38]. P4H acts as an oxygen sensor in cells and regulates the degradation of hypoxia-inducible factor (HIF) in proteasomes in an oxygen-dependent manner. HIF is activated by P4H hydroxylation and regulates adaptive hypoxic responses [39]. A study has shown the involvement of P4H in the pathogenesis of NAFLD [36]. Our results showed that *P4HA1* was a hub gene in NAFLD. Furthermore, a study has shown differential expression of *P4HA1* in the liver of patients with morbid obesity [40]. Our results revealed a decrease in *P4HA1* expression level in patients with NASH.

In the KEGG enrichment analysis, FOXO1 is involved in both the insulin signaling pathway, and FOXO1 was the characteristic target gene for NAFLD. A study showed that Lut/ZnO NPs could activate the PI3K/AKT signaling pathway, thereby inactivating *FOXO1*. Thus, Lut/ZnO NPs could alleviate NAFLD progression by reducing insulin resistance and antioxidant levels, as well as by regulating the insulin signaling pathway [41]. Furthermore, *LPL* was mainly enriched in chromosome segregation. A paradoxical response to DNA damage occurs in HCC, which leads to errors in chromosome segregation [42]. Error in mitosis occurs in metabolic disorders and causes numerical and structural chromosome aberrations during cell division [43]. *FMO1* and *P4HA1* involved in these pathways are closely associated with HCC [44]. Inflammation and ICI are critically involved in NALFD development. *P4HA1* was primarily enriched in the interaction between neuroactive ligand receptors was a close relationship of NALFD [45].

Activated innate immune cells are involved in NAFLD pathogenesis. In fact, NAFLD progression involves recognizing immune cells, such as Kupffer cells, neutrophils, DCs, and natural killer T cells, by pattern recognition. This leads to oxidative imbalance, which promotes the production of cytokines and new reactive species by innate immune cells, thereby promoting inflammation induced by adaptive immune cells. Recent studies suggest that the activation of innate and adaptive immune cells causes inflammation and fibrosis in the liver. Tfh cells cause dysregulation of humoral immunity in patients with liver cirrhosis [46] and are involved in virus-induced liver fibrosis [47]. The results of this study revealed a significant negative correlation between *AMDHD1* and Tfh cells. Therefore, it is speculated that *AMDHD1* could aid in preventing liver fibrosis and cirrhosis among patients with NAFLD. In addition, HSC activation induces liver fibrogenesis in NAFLD. CD4 + T cells suppressed Th9 cell differentiation and reduced IL-9 expression, thus promoting the activation of hepatic stellate cells (HSCs) by activating the Raf/MEK/ERK signaling pathway [48].

TFs and miRNAs regulate gene expression and its downstream targets. MiRNAs regulate genes associated with normal metabolism in the liver. MiRNA dysregulation is involved in the development and progression of NAFLD [49]. microRNA-432-5p suppresses *E2F3* translation by binding to the 3' UTR of *E2F3*, thereby influencing the invasion and migratory abilities of liver cancer cells [50]. miR-124-3p is involved in NAFLD development by directly targeting preadipocyte factor-1 [51]. miR-124-3p modulates sirtuin 1 expression in liver cancer, thereby attenuating the growth of liver cancer cells [52].

The results of this study showed that CTCF was regulated by *AMDHD1, P4HA1,* and *FMO1*. CTCF could alleviate NAFLD. Specific deletion of CTCF in the liver causes augments PPARγ-DNA binding activity, which increases downstream lipid MRG expression, thus causing hepatosteatosis [53].

### Study strengths and limitations

For the first time, MRBs in patients with NAFLD were identified using GEO to discover new targets for treating patients with NAFLD. In addition, these biomarkers and differential ICI would aid in exploring the correlation between NAFLD and the immune microenvironment. Nevertheless, the present study has a few limitations. First, this is a retrospective study, and the data were obtained from publicly available databases. Hence, the results should be validated using additional clinical samples and data. Second, the mechanism of action of these MRBs in NAFLD progression needs to be determined.

## Conclusion

In summary, four MRBs, including *AMDHD1*, *FMO1*, *LPL*, and *P4HA1,* were identified and demonstrated good ability in distinguishing patients with NAFLD from HCs. The results of this study would aid in determining the involvement of metabolism in the onset and development of NAFLD. This would help identify new targets for diagnosing and treating patients with NAFLD.

### Supplementary Information

**Additional file 1.**

**Additional file 2.**

## Data Availability

The raw data of this study are derived from the TCGA database (https://portal.gdc.cancer.gov/) and the GEO data portal (https://www.ncbi.nlm.nih.gov/geo/), which are publicly available databases.

## Abbreviation

NAFLD: Non-alcoholic fatty liver disease
NASH: Nonalcoholic steatohepatitis
HC: Healthy control
GEO: Gene Expression Omnibus
DEGs: Differentially expressed genes
MRGs: Metabolism-related genes
MR-DEGs: Metabolism-related differentially expressed genes
PPI: Protein–protein interaction
LASSO: Least absolute shrinkage and selection operator
RF: Random forest
GO: Gene ontology
KEGG: Kyoto Encyclopedia of Genes and Genomes
ROC: Receiver operating characteristic
GSEA: Gene set enrichment analysis
TF: Transcription factor
qRT-PCR: Quantitative reverse transcriptase PCR
