# Supplementary material for: Identification of metabolic biomarkers associated with nonalcoholic fatty liver disease
Source: Lipids Health Dis. 2023 Sep 11;22:150. doi: 10.1186/s12944-023-01911-2 (PMC10494330; doi:10.1186/s12944-023-01911-2)
Supplement: Supplementary file 1 — Additional file 1. [file 12944_2023_1911_MOESM1_ESM.doc]

Supplementary Table 1 The information of the included participants.

| Parameter | HC（n=5） | Nash（n=5） | P |  |
| --- | --- | --- | --- | --- |
| Age（years） | 49±8.46 | 46.60±7.50 | 0.648 |  |
| Sex（M：F） | 3:2 | 2:3 | 1.00 |  |
| Race（Han HA）  Diabetes mellitus  Hypertension  TC（mmol/L） | 5  1  2  6.39±3.14 | 5  3  3  15.77±3.32 | 0.524  1.00  0.002 |  |
| TG（mmol/L） | 6.56±5.18 | 11.34±6.17 | 0.221 |  |
| LDL-C(mmol/L) | 0.67±0.22 | 0.60±0.10 | 0.520 |  |
| HDL-C(mmol/L) | 2.73±1.06 | 3.09±1.62 | 0.690 |  |
| ALT(U/L) | 26.00±9.46 | 47.20±20.66 | 0.070 |  |
| AST(U/L) | 35±14.98 | 44.4±14.40 | 0.341 |  |
| GGT(U/L) | 31.20±14.65 | 66.80±33.74 | 0.062 |  |
| BMI | 24.78±0.96 | 28.49±1.83 | 0.007 |  |
| WC(cm) | 74.70±13.81 | 85.67±9.96 | 0.188 |  |

Data are presented as the mean ± standard deviation. *P < 0.05. All clinical and

|  |
| --- |

demographic data were analyzed using the unpaired t-test with an exception of “sex” ,

“Race”，“Diabetes mellitus”and“Hypertension”, which were analyzed with

The Exact probability method of four cell table. Abbreviations:HC,Health Control；ALT, alanine aminotransferase;AST, aspartate aminotransferase; BMI, body mass index; GGT, gamma glutamyl trans-peptidase; HDL-C, high-density lipoprotein cholesterol; LDL-C, low-density lipoprotein cholesterol; TC, total cholesterol; TG, total triglycerides; WC, waist circumference.

|  |  |  |  |  |  |  |  |  |  |  |
| --- | --- | --- | --- | --- | --- | --- | --- | --- | --- | --- |
|  |  |  |  |  |  |  |  |  |  |  |
|  |  |  |  |  |  |  |  |  |  |  |
|  |  |  |  |  |  |  |  |  |  |  |
|  |  |  |  |  |  |  |  |  |  |  |
|  |  |  |  |  |  |  |  |  |  |  |
