# Supplementary material for: Identification of metabolic biomarkers associated with nonalcoholic fatty liver disease
Source: Lipids Health Dis. 2023 Sep 11;22:150. doi: 10.1186/s12944-023-01911-2 (PMC10494330; doi:10.1186/s12944-023-01911-2)
Supplement: Supplementary file 2 — Additional file 2. [file 12944_2023_1911_MOESM2_ESM.docx]

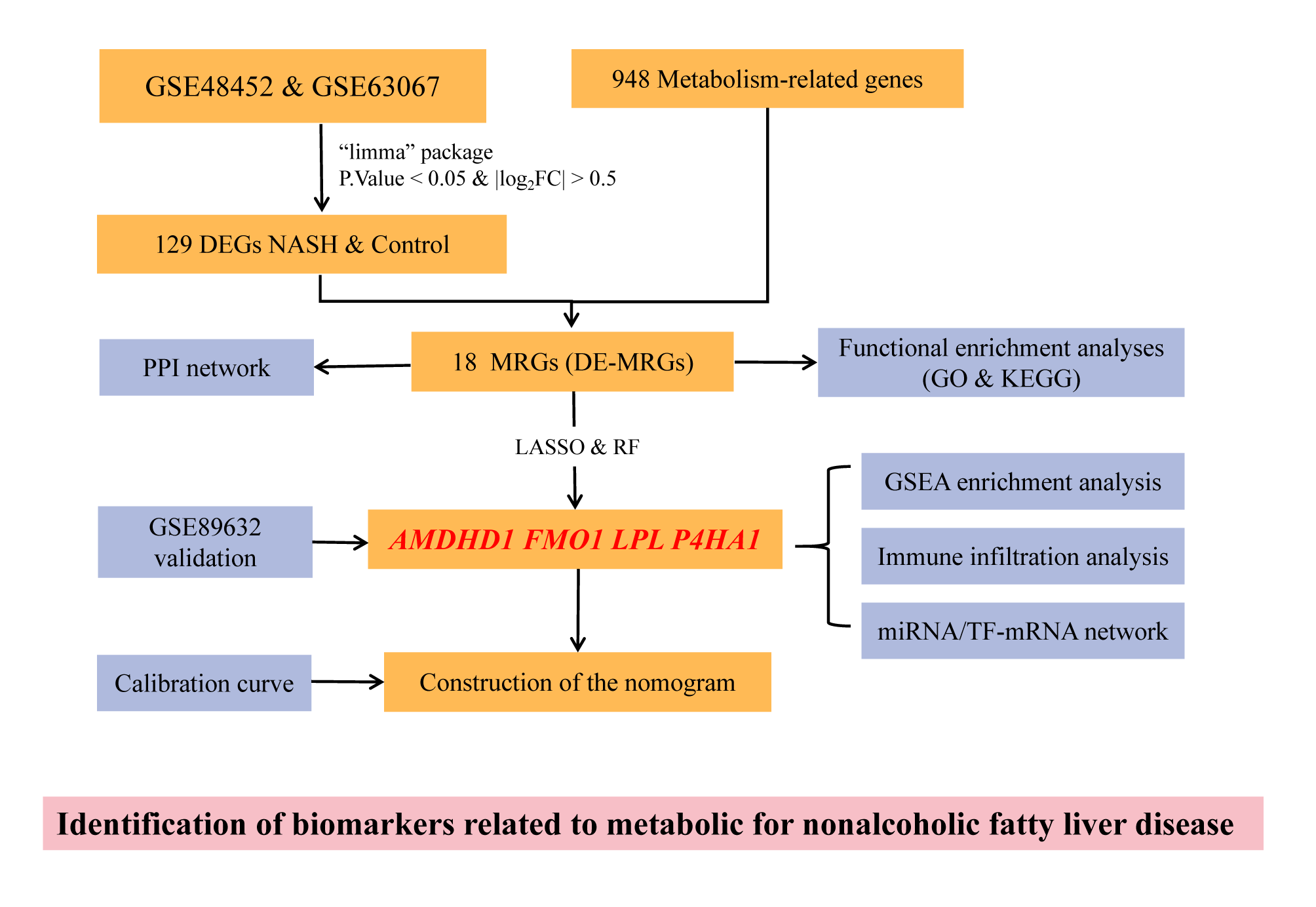


**Supplementary Figure 1** The flow chart of this study.


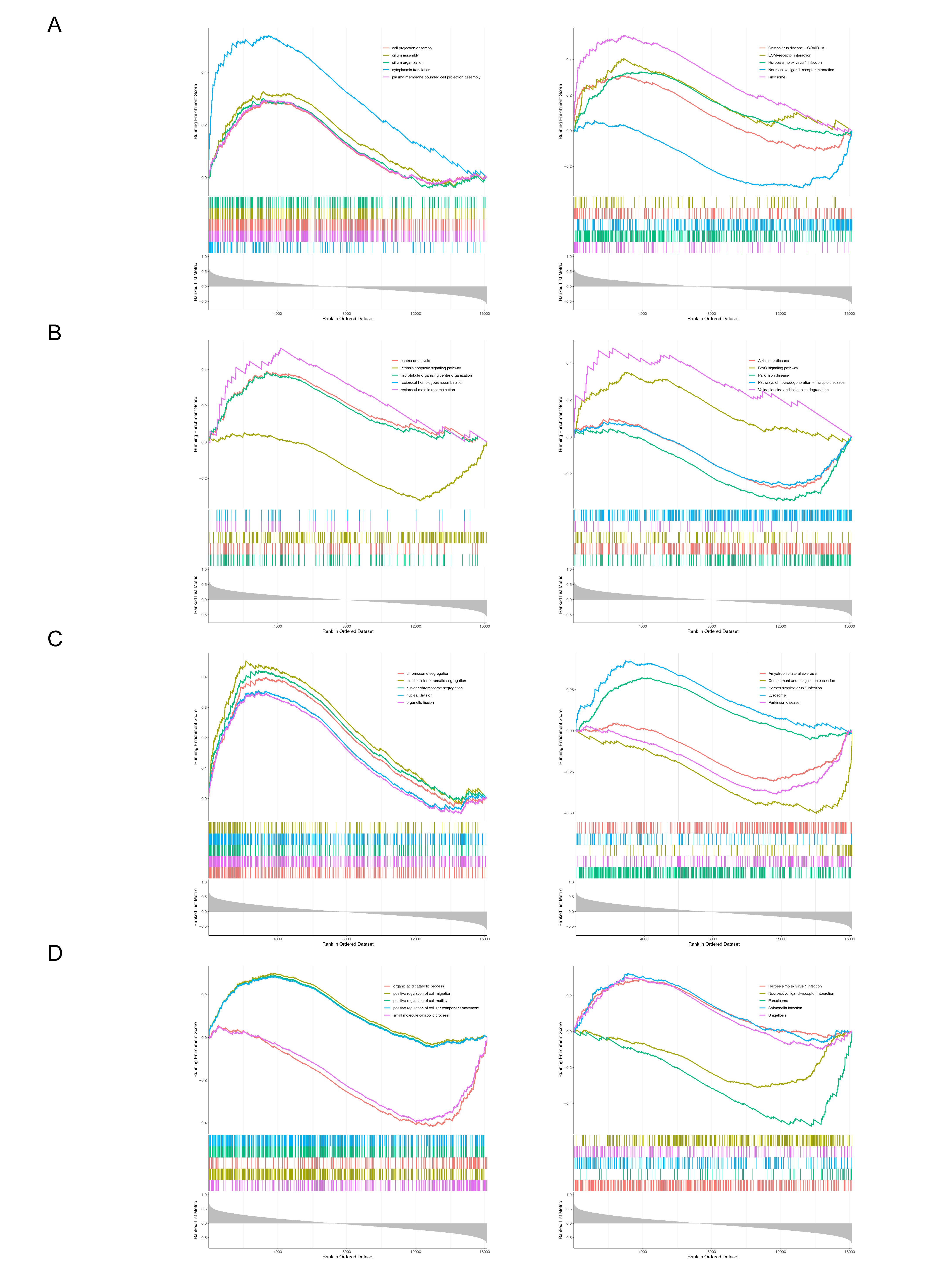


**Supplementary Figure 2** The potential KEGG pathways associated with biomarkers A, AMDHD1 B, *FMO1* C, *LPL* D, *P4HA1*.
